# Pevonedistat in East Asian patients with acute myeloid leukemia or myelodysplastic syndromes: a phase 1/1b study to evaluate safety, pharmacokinetics and activity as a single agent and in combination with azacitidine

**DOI:** 10.1186/s13045-022-01264-w

**Published:** 2022-05-11

**Authors:** Hiroshi Handa, June-Won Cheong, Yasushi Onishi, Hiroatsu Iida, Yukio Kobayashi, Hyeoung-Joon Kim, Tzeon-Jye Chiou, Koji Izutsu, Olga Tsukurov, Xiaofei Zhou, Helene Faessel, Ying Yuan, Farhad Sedarati, Douglas V. Faller, Akiko Kimura, Shang-Ju Wu

**Affiliations:** 1 National University Corporation, Gunma University, Maebashi, Japan; 2 Severence Hospital, Yonsei University Health System, Seoul, South Korea; 3 National University, Corporation Tohoku University, Sendai, Japan; 4 National Hospital Organization Nagoya Medical Center, Nagoya, Japan; 5 International University of Health and Welfare, Mita Hospital, Tokyo, Japan; 6 Chonnam National University, Hwasun Hospital, Hwasun, South Korea; 7 Taipei Municipal Wanfang Hospital, Taipei Medical University, Taipei Veterans General Hospital, Taipei, Taiwan; 8 National Cancer Center Hospital, Tokyo, Japan; 9 Takeda Development Center Americas, Inc. (TDCA), Lexington, MA USA; 10 Takeda Pharmaceutical Company Limited, Osaka, Japan; 11 Division of Hematology, Department of Internal Medicine, Zhongzheng Dist, National Taiwan University Hospital, No.7, Zhongshan S. Rd., Taipei City, 100 Taiwan

**Keywords:** AML, MDS, Pevonedistat, Phase 1/1b, East Asian

## Abstract

Pevonedistat, the first small-molecule inhibitor of NEDD8-activating enzyme, has demonstrated clinical activity in Western patients with acute myeloid leukemia (AML) and myelodysplastic syndromes (MDS). We report findings from a phase 1/1b study in East Asian patients with AML or MDS, conducted to evaluate the safety/tolerability and characterize the pharmacokinetics of pevonedistat, alone or in combination with azacitidine, in this population, and determine the recommended phase 2/3 dose for pevonedistat plus azacitidine. Twenty-three adult patients with very high/high/intermediate-risk AML or MDS were enrolled in Japan, South Korea and Taiwan. All 23 patients experienced at least one grade ≥ 3 treatment-emergent adverse event. One patient in the combination cohort reported a dose-limiting toxicity. Eighteen patients discontinued treatment; in nine patients, discontinuation was due to progressive disease. Three patients died on study of causes considered unrelated to study drugs. Pevonedistat exhibited linear pharmacokinetics over the dose range of 10–44 mg/m^2^, with minimal accumulation following multiple-dose administration. An objective response was achieved by 5/11 (45%) response-evaluable patients in the pevonedistat plus azacitidine arm (all with AML), and 0 in the single-agent pevonedistat arm. This study showed that the pharmacokinetic and safety profiles of pevonedistat plus azacitidine in East Asian patients were similar to those observed in Western patients as previously reported. The recommended Phase 2/3 dose (RP2/3D) of pevonedistat was determined to be 20 mg/m^2^ for co-administration with azacitidine 75 mg/m^2^ in Phase 2/3 studies, which was identical to the RP2/3D established in Western patients.

*Trial registration*: clinicaltrials.gov: NCT02782468 25 May 2016. https://clinicaltrials.gov/ct2/show/NCT02782468

**Supplementary Information:**

The online version contains supplementary material available at 10.1186/s13045-022-01264-w.

## To the Editor

There is a critical unmet need for novel treatment options that can improve outcomes in patients with acute myeloid leukemia (AML) or higher-risk myelodysplastic syndromes (MDS). Pevonedistat (MLN4924; TAK-924) is an investigational small-molecule inhibitor of neural precursor cell expressed, developmentally downregulated 8 (NEDD8)-activating enzyme (NAE) [1–3]. Upregulation of the NEDD8 cascade is associated with cancer pathogenesis, making it a compelling target for drug development [4, 5]. Pevonedistat forms an adduct with NAE, preventing activation of the cascade and ultimately leading to substrate accumulation and cell death. A phase 1b study of pevonedistat and azacitidine combination treatment conducted in Western patients aged ≥ 60 years with untreated AML showed that the combination was well tolerated and exhibited clinical activity, with an objective response rate (ORR) of 50% [6]. The recommended phase 2/3 dose (RP2/3D) of pevonedistat for co-administration with azacitidine was determined to be 20 mg/m^2^.

Pharmacokinetics (PK) can differ between Asian and Western patients. We conducted an open-label phase 1/1b dose escalation/expansion study (NCT02782468) to assess the safety/tolerability and PK of pevonedistat as a single agent or in combination with azacitidine in East Asian patients with AML or higher-risk MDS, and to determine the RP2/3D for combination treatment in this population. Full study design and methods are provided in Additional file 1.

A total of 23 patients were enrolled in Japan, South Korea and Taiwan (*n* = 12/4/7). Ten patients received single-agent pevonedistat 25 mg/m^2^ or 44 mg/m^2^ (*n* = 3/7), and 13 patients received pevonedistat 10 mg/m^2^ or 20 mg/m^2^ (*n* = 3/10) plus azacitidine 75 mg/m^2^. Patient demographics and disease characteristics are shown in Additional file 1: Table S1. At data cut-off, 5 patients remained on combination treatment, while 18 had discontinued study treatment, primarily due to progressive disease (PD; *n* = 9) or adverse events (AEs; *n* = 6).

## Safety

All 23 patients experienced at least one grade ≥ 3 treatment-emergent AE (TEAE). The safety profile of pevonedistat with/without azacitidine in East Asian patients (summarized in Table 1) was comparable to that in Western patients [6, 7], with the most common TEAEs including constipation, nausea, and anemia (Additional file 1: Table S2).

**Table 1:** Overall summary of TEAEs (safety population)

	Pevonedistat			Pevonedistat + azacitidine 75 mg/m^2^			Total
	25 mg/m^2^ *N* = 3 *n* (%)	44 mg/m^2^ *N* = 7 *n* (%)	Total *N* = 10 *n* (%)	10 mg/m^2^ *N* = 3 *n* (%)	20 mg/m^2^ *N* = 10 *n* (%)	Total *N* = 13 *n* (%)	*N* = 23 *n* (%)
*Any TEAE*	3 (100)	7 (100)	10 (100)	3 (100)	10 (100)	13 (100)	23 (100)
Grade ≥ 3^a^	3 (100)	7 (100)	10 (100)	3 (100)	10 (100)	13 (100)	23 (100)
Grade ≥ 4^a^	0	2 (28)	2 (20)	2 (66)	5 (50)	7 (53)	9 (39)
*Any drug-related TEAE*	3 (100)	3 (43)	6 (60)	3 (100)	8 (80)	11 (85)	17 (74)
Grade ≥ 3^a^	2 (67)	2 (29)	4 (40)	3 (100)	7 (70)	10 (76)	14 (61)
SAE	2 (67)	7 (100)	9 (90)	2 (67)	4 (40)	6 (46)	15 (65)
Drug-related SAE	0	1 (14)	1 (10)	1 (33)	2 (20)	3 (23)	4 (17)
TEAEs resulting in study drug discontinuation	0	2 (29)	2 (20)	2 (67)	1 (10)	3 (23)	5 (22)
TEAEs resulting in discontinuation from the study	0	3 (43)	3 (30)	2 (67)	2 (20)	4 (31)	7 (30)
On-study deaths^b^	0	1 (14)	1 (10)	1 (33)	1 (10)	2 (15)	3^**c**^ (13)

SAE, serious adverse event; TEAE, treatment-emergent adverse event

TEAE was defined as any adverse event that occurred after administration of the first dose of study treatment and up through 30 days after the last dose of study drug, any event that was considered drug related regardless of the start date of the event, or any event that was present at baseline but worsened in severity after baseline. Percentages are based on the total number of patients in safety population in each column. A patient counts once for each event

^a^Individual grades represent the maximum severity a patient experienced

^b^On-study deaths were defined as deaths that occurred between the first dose of study drug and 30 days after the last dose of study drug

^c^AML, pneumonia and acute kidney injury (*n* = 1 each); no on-study deaths were attributed to study treatments

Only one of twenty evaluable patients experienced DLTs: One patient receiving pevonedistat 20 mg/m^2^ plus azacitidine experienced grade 3 atrial fibrillation and grade 3 tumor lysis syndrome. The RP2/3D of pevonedistat in combination with azacitidine was therefore determined to be 20 mg/m^2^.

## Pevonedistat PK

Pevonedistat PK data (summarized in Additional file 1: Table S3) showed that systemic exposure increased in an approximately dose-proportional manner over the 10–44 mg/m^2^ dose range. There was minimal accumulation following multiple-dose administration, consistent with the mean terminal disposition phase half-life (T_1/2z_) of approximately 8 h. Clearance rates were comparable for pevonedistat as a single agent and when co-administered with azacitidine, suggesting that co-administration has no clinically meaningful effects on pevonedistat exposure. These findings were consistent with previous analyses of pevonedistat PK in Western patients (Additional file 1: Table S4) [6–9].

## Antitumor activity

**Fig. 1:**
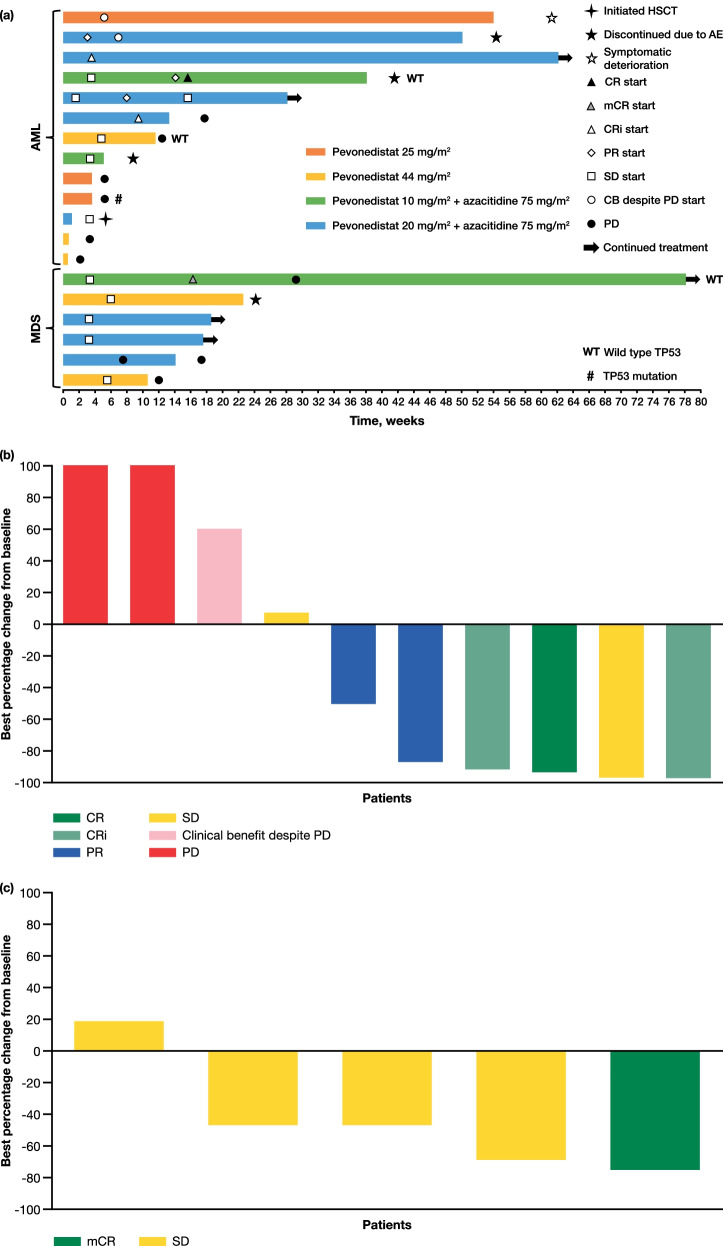
Treatment responses in the response-evaluable population^a^ (*N* = 19): **a** Swimmer plot showing responses and duration of treatment^b^; Best percentage change from baseline in myeloblast count in **b** patients with AML^c^ and **c** patients with MDS^d^. AE, adverse event; AML, acute myeloid leukemia; CB, clinical benefit; CR, complete remission; CRi, complete remission with incomplete blood count recovery; HSCT, hematopoietic stem cell transplant; mCR, marrow complete remission; MDS, myelodysplastic syndromes; PD, progressive disease; PR, partial remission; SD, stable disease. ^a^All patients who received at least one dose of study drug, had a baseline disease assessment, and had at least one post-baseline disease assessment. ^b^For patients who were ongoing treatment at data cut-off and who therefore did not have a date of last visit, their date of last assessment was used to determine bar length. *TP53* mutation status is indicated for the 4 patients with available data. Mutation status was unknown in the remaining patients. ^c^Two patients with AML in the single-agent pevonedistat 44 mg/m^2^ dose cohort and one patient with AML in the pevonedistat 10 mg/m^2^ combination arm dose cohort were excluded due to insufficient bone marrow aspirate blast data. The patient with AML with a decrease in blast count and stable disease had an abnormal cytogenetic finding at screening; stable disease was recorded on cycle 1 day 15 on November 27, 2017, and lasted to the end of study on December 7, 2017. The patient discontinued the study to initiate a hematopoietic stem cell transplant. ^d^One patient with MDS in the pevonedistat 20 mg/m^2^ combination arm dose cohort was excluded due to insufficient bone marrow aspirate blast data

Treatment duration and responses for the 19 evaluable patients are illustrated in Fig. 1a. ORRs were 0% in the single-agent pevonedistat arm (*N* = 8) and 45% in the pevonedistat plus azacitidine arm (*N* = 11; Additional file 1: Table S5). The median duration of response in the 5 responding patients, all of whom had AML, was 4.8 months (range 1–14 months) at data cut-off. The majority of patients with MDS (*N* = 6) had stable disease (*n* = 4), while one achieved marrow complete remission (mCR) and one had PD (Additional file 1: Table S5). Changes from baseline in myeloblast count in patients with AML and MDS are shown in Fig. 1b, c.

In summary, the safety and PK profiles of pevonedistat in East Asian patients were consistent with those seen in Western patients. Clinical activity was demonstrated, with an ORR of 45% in East Asian patients with AML treated with pevonedistat plus azacitidine. The RP2/3D for pevonedistat in combination with azacitidine was 20 mg/m^2^, the same as that previously determined in Western patients [6]. This supports use of the same treatment regimens in Western and East Asian patients in future global trials, which may help expedite access to pevonedistat-based treatment in Asia.

## Supplementary Information

**Additional file 1**. Supplementary methods, tables, and figure.

## Data Availability

The datasets, including the redacted study protocol, redacted statistical analysis plan and individual participants data supporting the results reported in this article, will be made available within 3 months from initial request, to researchers who provide a methodologically sound proposal. The data will be provided after its de-identification, in compliance with applicable privacy laws, data protection, and requirements for consent and anonymization.

## Abbreviations

AE: Adverse event
(Allo-)HSCT: (Allogeneic) hematopoietic stem cell transplantation
AML: Acute myeloid leukemia
CR: Complete remission
CRi: Complete remission with incomplete blood count recovery
DLT: Dose-limiting toxicity
HI: Hematologic improvement
mCR: Marrow complete remission
MDS: Myelodysplastic syndromes
NAE: NEDD8-activating enzyme
NEDD8: Neural precursor cell expressed developmentally down-regulated protein 8
ORR: Objective response rate
PD: Progressive disease (disease progression)
PK: Pharmacokinetic(s)
PR: Partial remission
RP2/3D: Recommended phase 2/phase 3 dose
RRM2: Ribonucleotide reductase
SAE: Serious adverse event
SD: Stable disease
T_1/2z_: Terminal disposition phase half-life
TEAE: Treatment-emergent adverse event
