# Supplementary material for: Pevonedistat in East Asian patients with acute myeloid leukemia or myelodysplastic syndromes: a phase 1/1b study to evaluate safety, pharmacokinetics and activity as a single agent and in combination with azacitidine
Source: J Hematol Oncol. 2022 May 11;15:56. doi: 10.1186/s13045-022-01264-w (PMC9097234; doi:10.1186/s13045-022-01264-w)

**Supplementary information**

**Article tile:**

Pevonedistat in East Asian patients with acute myeloid leukemia or myelodysplastic syndromes: A phase 1/1b study to evaluate safety, pharmacokinetics and activity as a single agent and in combination with azacitidine

**Authors:**

Hiroshi Handa, June-Won Cheong, Yasushi Onishi, Hiroatsu Iida, Yukio Kobayashi, Hyeoung-Joon Kim, Tzeon-Jye Chiou, Koji Izutsu, Olga Tsukurov, Xiaofei Zhou, Helene Faessel, Ying Yuan, Farhad Sedarati, Douglas V. Faller, Akiko Kimura, Shang-Ju Wu

**Additional file 1:**

## Methods

### Study design

This multicenter, open-label, phase 1/1b dose-escalation and expansion study of pevonedistat alone or in combination with azacitidine was conducted at four centers in Japan, three in South Korea, and two in Taiwan. The study used a 3+3 design in the dose‑escalation phase, followed by cohort expansion to confirm the safety of single-agent pevonedistat and the RP2/3D of pevonedistat in combination with azacitidine **(Supplementary Fig. 1)**. Patients were initially enrolled to receive intravenous (IV) pevonedistat 25 mg/m^2^ on days 1, 3, and 5 in 21-day treatment cycles. If this dose was tolerated, patients were enrolled to two cohorts: single-agent IV pevonedistat 44 mg/m^2^ on days 1, 3, and 5 in 21-day cycles, or IV pevonedistat 10 mg/m^2^ on days 1, 3, and 5 in combination with IV or subcutaneous azacitidine 75 mg/m^2^ on days 1–5, 8, and 9 in 28-day treatment cycles. No further dose escalation was planned with single-agent pevonedistat; a second dose level of pevonedistat 20 mg/m^2^ was planned for the combination arm. No intrapatient dose escalation was permitted. The RP2/3D was determined as the highest dose level at which no more than one in a minimum of six patients experienced a dose-limiting toxicity (DLT) during cycle 1; DLTs included grade ≥3 nonhematologic toxicity considered by the investigator to be at least possibly related to pevonedistat or azacitidine.

***Patients***

Adult East Asian patients who had adequate hematologic, renal, hepatic, and cardiac function, and an Eastern Cooperative Oncology Group performance status (ECOG PS) of 0–1, were eligible. Patients with a confirmed diagnosis of World Health Organization (WHO)‑defined AML (including leukemia secondary to prior chemotherapy or resulting from an antecedent hematologic disorder) who had failed to achieve complete remission (CR) or had relapsed after prior therapy and were not candidates for potentially curative therapy, and patients with higher-risk MDS or chronic myelomonocytic leukemia (CMML) who met Revised International Prognostic Scoring System (IPSS-R) criteria for the very high, high, or intermediate risk groups and for whom standard curative or life-prolonging treatment was unavailable, were eligible for both the single-agent and combination arms. Additionally, patients aged ≥60 years with previously untreated AML who had <30% bone marrow blasts and who were not candidates for standard induction chemotherapy, and patients with previously untreated higher-risk MDS/CMML who met IPSS-R criteria for the very high, high, or intermediate risk groups, were eligible for the combination arm. Patients with acute promyelocytic leukemia or AML associated with t(9;22) karyotypes were excluded, as were patients eligible for HSCT or with known central nervous system involvement. Patients were not permitted to have received prior treatment with systemic antineoplastic therapy (except hydroxyurea), radiotherapy, or any investigational products within 14 days of the first dose of the study drug; or moderate or strong cytochrome P450 (CYP) 3A inhibitors or inducers within 7 days; or amiodarone within the previous 6 months. Patients who had received >3 prior lines of therapy or prior therapy with hypomethylating agents were excluded from the combination arm.

***Study objectives and assessments***

The study had three primary objectives. The first was to evaluate the safety and tolerability of single-agent pevonedistat in East Asian patients with R/R AML or higher‑risk MDS. The second was to evaluate the safety and tolerability and determine the RP2/3D of pevonedistat in combination with azacitidine in East Asian patients with AML or higher-risk MDS. The third was to characterize the PK of pevonedistat as a single agent or in combination with azacitidine. The secondary objective was to evaluate disease response. An exploratory objective was to evaluate the relationship between molecular characteristics at baseline, such as cytogenetic abnormalities, mutations in key genes used in risk determination in AML and MDS, and epigenetic characteristics, with overall response and/or safety.

Toxicities were recorded throughout the study and until 30 days after the last dose of pevonedistat or azacitidine. Adverse events (AEs) were graded according to the National Cancer Institute’s Common Terminology Criteria for AEs version 4.03. Blood samples for pevonedistat PK assessments were collected pre-dose, at the end of infusion, and at 1, 2, 4, 6, and 10 hours post-infusion on days 1 and 5, and at 24 and 48 hours post-dose (i.e. on days 2, 3, 6, and 7) during cycle 1. Actual PK sampling times were used in the derivation of PK parameters. Pevonedistat plasma concentrations were measured by validated high performance liquid chromatography with tandem mass spectrometry as previously reported (Sarantopoulos J, Shapiro GI, Cohen RB, Clark JW, Kauh JS, Weiss GJ, et al. Phase I study of the investigational NEDD8-activating enzyme inhibitor pevonedistat (TAK-924/MLN4924) in patients with advanced solid tumors. Clin Cancer Res. 2016;22(4):847–57). The dynamic range of the plasma assay was 1–500 ng/mL. Plasma concentration values below the lower limit of quantification of the bioanalytical assay were set to 0 for analysis.

Best overall response was assessed by investigators according to the International Working Group (IWG) criteria for AML or the modified IWG criteria for MDS/CMML (Cheson BD, et al. J Clin Oncol. 2003;21[24]:4642–9; Cheson BD, et al. Blood. 2006;108)2]:419–25). Bone marrow aspirates were collected during screening to analyze disease burden, and at the end of cycle 2 and cycle 4 (day 21 [-6 days] for the single-agent pevonedistat arm and any time between days 20 and 28 for the combination arm, provided that the disease assessment was available before day 1 of the following cycle) to assess disease response. After cycle 4, bone marrow aspirates were collected after completion of every third cycle (cycle 7, cycle 10, etc.) and at the end of study visit for patients who withdrew for reasons other than progressive disease (PD). Additional bone marrow aspirates could be performed if warranted by changes in peripheral blood counts. Bone marrow biopsies were conducted and bone marrow aspirate samples collected during screening to analyze cytogenetics and karyotype, and an additional non-mandatory sample was taken at screening for baseline molecular characterization (e.g. *NPM1*, *FLT3*, *CEBPA*, messenger RNA, and microRNA). Mutational profiling was performed centrally via targeted next generation sequencing (NGS) using a custom 572-gene panel containing common AML and MDS genes (Clinical Research Sequencing Platform, Broad Institute of MIT and Harvard, Cambridge, MA, USA). Nineteen genes most frequently mutated in MDS and AML (*ASXL1, CEBPA, DNMT3A, FLT3, IDH1, IDH2, NPM1, RUNX1, JAK2, KRAS, ETV6, EZH2, KIT, NRAS, SF3B1, SRSF2, TET2, TP53, and WT1*) were selected for this analysis. Local site reports were also collected for baseline mutations and cytogenetic abnormalities.

***Statistical analysis***

All statistical analyses were primarily descriptive in nature, and no formal statistical tests were performed. The DLT‑evaluable population was defined as patients who either experienced DLT during cycle 1 or received all scheduled doses of study drug in cycle 1 without DLT. All safety analyses were performed using the safety population, which was defined as all enrolled patients who received at least one dose of study drug. The PK-evaluable population was defined as all enrolled patients who had sufficient cycle 1 dosing and pevonedistat concentration–time data to reliably estimate PK parameters and who had not received any excluded concomitant medications. Plasma PK parameters of pevonedistat were determined from the concentration–time profiles of all evaluable patients by non-compartmental analysis using Phoenix WinNonLin (version 7; Pharsight Corporation, Cary, NC, USA). The response-evaluable population was defined as all patients who received at least one dose of study drug, had a baseline disease assessment, and had at least one post-baseline disease assessment.

**Supplementary Table 1** Baseline patient demographics and disease characteristics

|  | **Pevonedistat** | | | **Pevonedistat + azacitidine 75 mg/m^2^** | | | **Total** |
| --- | --- | --- | --- | --- | --- | --- | --- |
| **Characteristics** | **25 mg/m^2^**  ***N* = 3** | **44 mg/m^2^**  ***N* = 7** | **Total**  ***N* = 10** | **10 mg/m^2^**  ***N* = 3** | **20 mg/m^2^**  ***N* = 10** | **Total**  ***N* = 13** | ***N* = 23** |
| **Sex, *n* (%)** |  |  |  |  |  |  |  |
| Male | 2 (67) | 7 (100) | 9 (90) | 3 (100) | 7 (70) | 10 (77) | 19 (83) |
| Female | 1 (33) | 0 | 1 (10) | 0 | 3 (30) | 3 (23) | 4 (17) |
| **Age (years)^a^** |  |  |  |  |  |  |  |
| *n* | 3 | 7 | 10 | 3 | 10 | 13 | 23 |
| Mean (StD) | 78.0 (5.20) | 69.0 (11.27) | 71.7 (10.47) | 65.0 (9.54) | 64.9 (8.52) | 64.9 (8.34) | 67.9 (9.72) |
| Median | 75.0 | 71.0 | 74.5 | 60.0 | 66.5 | 65.0 | 69.0 |
| Min, max | 75, 84 | 47, 83 | 47, 84 | 59, 76 | 45, 74 | 45, 76 | 45, 84 |
| **BSA (m^2^)^b^** |  |  |  |  |  |  |  |
| *n* | 3 | 7 | 10 | 3 | 10 | 13 | 23 |
| Mean (StD) | 1.5 (0.26) | 1.7 (0.21) | 1.7 (0.24) | 1.7 (0.04) | 1.7 (0.18) | 1.7 (0.16) | 1.7 (0.20) |
| Median | 1.6 | 1.7 | 1.7 | 1.7 | 1.7 | 1.7 | 1.7 |
| Min, max | 1, 2 | 1, 2 | 1, 2 | 2, 2 | 1, 2 | 1, 2 | 1, 2 |
| **Disease type, n (%)** |  |  |  |  |  |  |  |
| De novo AML | 2 (67) | 2 (40) | 4 (50) | 1 (50) | 4 (57) | 5 (56) | 9 (53) |
| Secondary AML | 1 (33) | 3 (60) | 4 (50) | 1 (50) | 3 (43) | 4 (44) | 8 (47) |
| AML secondary to prior existing MDS | 0 | 3 (60) | 3 (38) | 0 | 2 (29) | 2 (22) | 5 (29) |
| AML related to prior antineoplastic therapy | 0 | 0 | 0 | 1 (50) | 0 | 1 (11) | 1 (6) |
| Missing | 1 | 0 | 1 | 0 | 1 | 1 | 2 |
| De novo MDS | 0 | 2 (100) | 2 (100) | 1 (100) | 2 (67) | 3 (75) | 5 (83) |
| Secondary MDS | 0 | 0 | 0 | 0 | 1 (33) | 1 (25) | 1 (17) |
| MDS related to prior antineoplastic therapy | 0 | 0 | 0 | 0 | 1 (33) | 1 (25) | 1 (17) |

AML, acute myeloid leukemia; BSA, body surface area; max, maximum; MDL, myelodysplastic syndromes; min, minimum; StD, standard deviation

Percentages are based on the total number of patients in the safety population in each column

^a^Age at date of informed consent. ^b^BSA (m^2^) was calculated as [height (cm) × weight (kg) / 3600]^½^ based on the height and weight collected at screening. If a weight at screening was not available, then cycle 1 day 1 pre-dose weight was used

**Supplementary Table 2** Most common TEAEs (≥10% of patients) and grade ≥3 TEAEs (≥20% of patients) (safety population)

|  | **Pevonedistat** | | | **Pevonedistat + azacitidine 75 mg/m^2^** | | | **Total** |
| --- | --- | --- | --- | --- | --- | --- | --- |
| **Most common TEAEs**  **(≥10% of total patients)**  **Preferred term^a^** | **25 mg/m^2^**  ***N* = 3**  ***n* (%)** | **44 mg/m^2^**  ***N* = 7**  ***n* (%)** | **Total**  ***N* = 10**  ***n* (%)** | **10 mg/m^2^**  ***N* = 3**  ***n* (%)** | **20 mg/m^2^**  ***N* = 10**  ***n* (%)** | **Total**  ***N* = 13**  ***n* (%)** | ***N* = 23**  ***n* (%)** |
| Constipation | 1 (33) | 3 43) | 4 (40) | 2 (67) | 7 (70) | 9 (69) | 13 (57) |
| Nausea | 1 (33) | 3 (43) | 4 (40) | 1 (33) | 4 (40) | 5 (38) | 9 (39) |
| Pneumonia | 1 (33) | 6 (86) | 7 (20) | 1 (33) | 1 (10) | 2 (15) | 9 (39) |
| Vomiting | 1 (33) | 1 (14) | 2 (20) | 1 (33) | 6 (60) | 7 (54) | 9 (39) |
| Febrile neutropenia | 2 (67) | 4 (57) | 6 (60) | 0 | 2 (20) | 2 (15) | 8 (35) |
| Stomatitis | 0 | 4 (57) | 4 (40) | 1 (33) | 3 (30) | 4 (31) | 8 (35) |
| Diarrhea | 1 (33) | 2 (29) | 3 (30) | 0 | 4 (40) | 4 (31) | 7 (30) |
| Aspartate aminotransferase increased | 1 (33) | 1 (14) | 2 (20) | 1 (33) | 3 (30) | 4 (31) | 6 (26) |
| Back pain | 0 | 0 | 0 | 3 (100) | 3 (30) | 6 (46) | 6 (26) |
| Fatigue | 1 (33) | 2 (29) | 3 (30) | 2 (67) | 1 (10) | 3 (23) | 6 (26) |
| Insomnia | 1 (33) | 3 (43) | 4 (40) | 1 (33) | 1 (10) | 2 (15) | 6 (26) |
| Abdominal distention | 0 | 2 (29) | 2 (20) | 1 (33) | 2 (20) | 3 (23) | 5 (22) |
| Alanine aminotransferase increased | 1 (33) | 0 | 1 (10) | 1 (33) | 3 (30) | 4 (31) | 5 (22) |
| Anemia | 0 | 0 | 0 | 2 (67) | 3 (30) | 5 (38) | 5 (22) |
| Dizziness | 1 (33) | 1 (14) | 2 (20) | 1 (33) | 2 (20) | 3 (23) | 5 (22) |
| Dyspnea | 0 | 2 (29) | 2 (20) | 1 (33) | 2 (20) | 3 (23) | 5 (22) |
| Hypokalemia | 0 | 2 (29) | 2 (20) | 1 (33) | 2 (20) | 3 (23) | 5 (22) |
| Hypophosphatemia | 0 | 3 (43) | 3 (30) | 0 | 2 (20) | 2 (15) | 5 (22) |
| Platelet count decreased | 0 | 1 (14) | 1 (10) | 1 (33) | 3 (30) | 4 (31) | 5 (22) |
| Headache | 0 | 1 (14) | 1 (10) | 0 | 3 (30) | 3 (23) | 4 (17) |
| Hyperkalemia | 0 | 0 | 0 | 1 (33) | 3 (30) | 4 (31) | 4 (17) |
| Myalgia | 0 | 1 (14) | 1 (10) | 1 (33) | 2 (20 | 3 (23) | 4 (17) |
| Neutrophil count decreased | 0 | 0 | 0 | 1 (33) | 3 (30) | 4 (31) | 4 (17) |
| Pruritus | 0 | 0 | 0 | 2 (67) | 2 (20) | 4 (31) | 4 (17) |
| Pyrexia | 0 | 1 (14) | 1 (10) | 0 | 3 (30) | 3 (23) | 4 (17) |
| Rhinorrhea | 0 | 1 (14) | 1 (10) | 1 (33) | 2 (20) | 3 (23) | 4 (17) |
| Weight decreased | 0 | 2 (29) | 2 (20) | 0 | 2 (20) | 2 (15) | 4 (17) |
| Abdominal pain | 0 | 0 | 0 | 2 (67) | 1 (10) | 3 (23) | 3 (13) |
| Arthralgia | 0 | 2 (29) | 2 (20) | 1 (33) | 0 | 1 (8) | 3 (13) |
| Cough | 0 | 1 (14) | 1 (10) | 1 (33) | 1 (10) | 2 (15) | 3 (13) |
| Hyperuricemia | 0 | 1 (14) | 1 (10) | 0 | 2 (20) | 2 (15) | 3 (13) |
| Hypomagnesemia | 0 | 1 (14) | 1 (10) | 1 (33) | 1 (10) | 2 (15) | 3 (13) |
| Malaise | 0 | 2 (29) | 2 (20) | 1 (33) | 0 | 1 (8) | 3 (13) |
| Edema peripheral | 0 | 1 (14) | 1 (10) | 1 (33) | 1 (10) | 2 (15) | 3 (13) |
| Oropharyngeal pain | 0 | 1 (14) | 1 (10) | 0 | 2 (20) | 2 (15) | 3 (13) |
| Phlebitis | 0 | 1 (14) | 1 (10) | 1 (33) | 1 (10) | 2 (15) | 3 (13) |
| Pleural effusion | 0 | 3 (43) | 3 (30) | 0 | 0 | 0 | 3 (13) |
| Rash popular | 0 | 0 | 0 | 2 (67) | 1 (10) | 3 (23) | 3 (13) |
| Tumor lysis syndrome | 0 | 2 (29) | 2 (20) | 0 | 1 (10) | 1 (8) | 3 (13) |
| Upper respiratory tract infection | 0 | 1 (14) | 1 (10) | 1 (33) | 1 (10) | 2 (15) | 3 (13) |
| White blood cell decreased | 0 | 0 | 0 | 1 (33) | 2 (20) | 3 (23) | 3 (13) |
| **Grade ≥3 TEAEs (all patients)**  **Primary system organ class**  **High-level term**  **Preferred term^a^** |  |  |  |  |  |  |  |
| Patients with at least 1 | 3 (100) | 7 (100) | 10 (100) | 3 (100) | 10 (100) | 13 (100) | 23 (100) |
| Blood and lymphatic system disorders | 2 (67) | 5 (71) | 7 (70) | 2 (67) | 5 (50) | 7 (54) | 14 (61) |
| Neutropenia | 2 (67) | 4 (57) | 6 (60) | 0 | 4 (40) | 4 (31) | 10 (43) |
| Febrile neutropenia | 2 (67) | 4 (57) | 6 (60) | 0 | 2 (20) | 2 (15) | 8 (35) |
| Anemia | 0 | 0 | 0 | 2 (67) | 2 (20) | 4 (31) | 4 (17) |
| Leukocytosis | 0 | 2 (29) | 2 (20) | 0 | 0 | 0 | 2 (9) |
| Disseminated intravascular coagulation | 1 (33) | 0 | 1 (10) | 0 | 0 | 0 | 1 (4) |
| Bone marrow failure | 0 | 0 | 0 | 1 (33) | 0 | 1 (8) | 1 (4) |
| Infections and infestations | 2 (67) | 4 (57) | 6 (60) | 1 (33) | 3 (30) | 4 (31) | 10 (43) |
| Lower respiratory tract and lung infections | 1 (33) | 4 (57) | 5 (50) | 1 (33) | 1 (10) | 2 (15) | 7 (30) |
| Pneumonia | 1 (33) | 4 (57) | 5 (50) | 1 (33) | 1 (10) | 2 (15) | 7 (30) |
| Aspergillus infections | 1 (33) | 0 | 1 (10) | 0 | 1 (10) | 1 (8) | 2 (9) |
| Aspergillus infection | 0 | 0 | 0 | 0 | 1 (10) | 1 (8) | 1 (4) |
| Bronchopulmonary aspergillosis | 1 (33) | 0 | 1 (10) | 0 | 0 | 0 | 1 (4) |
| Urinary tract infection | 0 | 0 | 0 | 1 (33) | 0 | 1 (8) | 1 (4) |
| Investigations | 0 | 1 (14) | 1 (10) | 1 (33) | 4 (40) | 5 (38) | 6 (26) |
| Liver function analysis | 0 | 0 | 0 | 0 | 2 (20) | 2 (15) | 2 (9) |
| Alanine aminotransferase increased | 0 | 0 | 0 | 0 | 1 (10) | 1 (8) | 1 (4) |
| Gamma glutamyltransferase increased | 0 | 0 | 0 | 0 | 1 (10) | 1 (8) | 1 (4) |
| Platelet count decreased | 0 | 1 (14) | 1 (10) | 1 (33) | 0 | 1 (8) | 2 (9) |
| White blood cell analyses | 0 | 0 | 0 | 0 | 2 (20) | 2 (15) | 2 (9) |
| Neutrophil count decreased | 0 | 0 | 0 | 0 | 2 (20) | 2 (15) | 2 (9) |
| Metabolism and nutrition disorders | 0 | 3 (43) | 3 (30) | 1 (33) | 1 (10) | 2 (15) | 5 (22) |
| Electrolyte imbalance NEC | 0 | 2 (29) | 2 (20) | 0 | 1 (10) | 1 (8) | 3 (13) |
| Tumor lysis syndrome | 0 | 2 (29) | 2 (20) | 0 | 1 (10) | 1 (8) | 3 (13) |
| Hypophosphatemia | 0 | 2 (29) | 2 (20) | 0 | 0 | 0 | 2 (9) |
| Hypercalcemia | 0 | 1 (14) | 1 (10) | 0 | 0 | 0 | 1 (4) |
| Hyperglycemia | 0 | 1 (14) | 1 (10) | 0 | 0 | 0 | 1 (4) |
| Hypomagnesemia | 0 | 0 | 0 | 1 (33) | 0 | 1 (8) | 1 (4) |
| Hypokalemia | 0 | 0 | 0 | 1 (33) | 0 | 1 (8) | 1 (4) |
| Back pain | 0 | 0 | 0 | 0 | 1 (10) | 1 (8) | 1 (4) |
| Neoplasms benign, malignant and unspecified (including cysts and polyps) | 0 | 1 (14) | 1 (10) | 1 (33) | 0 | 1 (8) | 2 (9) |
| Malignant lung neoplasms | 0 | 0 | 0 | 1 (33) | 0 | 1 (8) | 1 (4) |
| Vascular disorders | 1 (33) | 1 (14) | 2 (20) | 0 | 0 | 0 | 2 (9) |
| Vascular hypertensive disorders | 1 (33) | 1 (14) | 2 (20) | 0 | 0 | 0 | 2 (9) |
| Hypertension | 1 (33) | 1 (14) | 2 (20) | 0 | 0 | 0 | 2 (9) |

PT, preferred term; TEAE, treatment-emergent adverse event

TEAE was defined as any adverse event that occurred after administration of the first dose of study treatment and up through 30 days after the last dose of study drug, any event that was considered drug related regardless of the start date of the event, or any event that was present at baseline but worsened in severity after baseline. Percentages are based on the total number of patients in the safety population in each column

^a^Patient counts once for each preferred term

**Supplementary Table 3** Key plasma PK parameters of pevonedistat following single- (day 1) and multiple-dose administration (day 5)

| **Plasma PK parameters^a^** | **Pevonedistat** | | | | **Pevonedistat + azacitidine 75 mg/m^2^** | | | |
| --- | --- | --- | --- | --- | --- | --- | --- | --- |
|  | **25 mg/m^2^** | | **44 mg/m^2^** | | **10 mg/m^2^** | | **20 mg/m^2^** | |
|  | Day | | Day | | Day | | Day | |
|  | 1 | 5 | 1 | 5 | 1 | 5 | 1 | 5 |
| *N* | 3 | 3 | 7 | 7 | 3 | 3 | 10 | 8 |
| C_max_ (ng/mL) | 253 (37.6) | 252 (9.9) | 410 (45.5) | 453 (38.7) | 67.0 (5.9) | 76.1 (17.6) | 160 (25.7) | 170 (33.7) |
| T_max_ ^b^ (h) | 0.87 (0.83; 0.92) | 0.85 (0.80; 1.00) | 1.00 (0.97; 1.22) | 1.00 (1.00; 1.02) | 1.10 (0.98; 1.15) | 1.00 (1.00; 1.15) | 1.00 (0.92; 1.12) | 1.00 (0.92; 1.13) |
| AUC_48_ (h*ng/mL) | 1491 (13.5) | 1515 (9.1) | 2282 (45.6) | 2163 (25.7) | 749 (51.8) | 647 (41.3) | 1182 (24.8) | 1224 (14.9) |
| T_1/2z_^c^ (h) | 7.80 (0.25) | 8.78 (1.86) | 7.67 (0.57) | 7.83 (0.88) | 7.28 (1.08) | 7.78 (2.769) | 8.98 (2.342) | 8.54 (2.530) |
| R_ac(AUC)_ | N/A | 1.02 (0.13) | N/A | 0.96 (0.16) | N/A | 0.869 (0.1261) | N/A | 1.08 (0.1176) |
| R_ac(Cmax)_ | N/A | N/A | N/A | N/A | N/A | 1.14 (0.1451) | N/A | 1.12 (0.1376) |
| CL (L/h) | 24.1 (20.1) | 24.0 (23.0) | 32.6 (41.7) | 34.8 (34.6) | 22.8 (48.8) | 28.1 (38.3) | 28.4 (23.9) | 28.9 (23.7) |
| Overall CL (L/h/m^2^) | 16.5 (11.0) | | 19.7 (28.9) | | 14.8 (39.2) | | 16.6 (21.0) | |
| V_ss_ (L) | 195 (32.9) | N/A | 260 (42.8) | N/A | 201 (37.3) | N/A | 292 (41.1) | N/A |

AUC_48_, area under the concentration-time curve from time 0 to 48 hours; AUC_τ_, area under the concentration-time curve during a dosing interval; CL, total clearance after intravenous administration; C_max_, maximum concentration (reported at the end of infusion even if the maximum observed concentration occurred at a later time point); NA, not applicable; Overall CL, CL on days 1 and 5 combined; PK, pharmacokinetic; R_ac(AUC)_, accumulation ratio based on AUC_τ_; R_ac(Cmax)_, accumulation ratio based on C_max_; T_1/2z_, terminal disposition phase half-life; T_max,_ time at which C_max_ occurred (end of infusion actual time); V_SS_, volume of distribution at steady state after intravenous administration

^a^Geometric mean (percent coefficient of variation); ^b^Mean (standard deviation)

**Supplementary Table 4** Comparison of key pevonedistat PK parameters at steady-state following IV administration of pevonedistat at 20 mg/m^2^ in combination with azacitidine

| **PK parameter (unit)** | **Asian patients from the present study (*N* = 8)** | **Western patients from study C15009^a^ (*N* = 26)** |
| --- | --- | --- |
| C_max_ (ng/mL) | 170 (34) | 163.7 (43) |
| AUC_0-48h_ (ng*h/mL) | 1224 (15) | 1099 (25) |
| T_1/2_ (h) | 8.54 (2.5) | 8.07 (2.1) |

^a^Swords RT et al. Blood. 2018 Mar 29;131(13):1415-1424

AUC_0-48h_; area under the plasma concentration-time curve from time 0 to 48 hours; C_max_, observed maximum plasma concentration; t_1/2_; terminal elimination half-life.

Parameters are presented as geometric mean (coefficient of variation%) except for t_1/2_ which is presented as mean (standard deviation).

**Supplementary Table 5** Summary of best response to treatment, by disease type (response-evaluable population^a^)

|  | **Pevonedistat** | | | **Pevonedistat + azacitidine 75 mg/m^2^** | | | **Total** |
| --- | --- | --- | --- | --- | --- | --- | --- |
|  | **25 mg/m^2^** | **44 mg/m^2^** | **Total** | **10 mg/m^2^** | **20 mg/m^2^** | **Total** |  |
| **AML** | ***N = 3*** | ***N = 3*** | ***N = 6*** | ***N = 2*** | ***N = 5*** | ***N = 7*** | ***N = 13*** |
| Best overall response, *n* (%) |  |  |  |  |  |  |  |
| CR | 0 | 0 | 0 | 1 (50) | 2 (40) | 3 (43) | 3 (23) |
| CR | 0 | 0 | 0 | 1 (50) | 0 | 1 (14) | 1 (8) |
| CRi | 0 | 0 | 0 | 0 | 2 (40) | 2 (29) | 2 (15) |
| PR | 0 | 0 | 0 | 0 | 2 (40) | 2 (29) | 2 (15) |
| SD | 0 | 1 (33) | 1 (17) | 1 (50) | 1 (20) | 2 (29) | 3 (23) |
| Clinical benefit rate despite PD | 1 (33) | 0 | 1 (17) | 0 | 0 | 0 | 1 (8) |
| PD^b^ | 2 (67) | 2 (67) | 4 (67) | 0 | 0 | 0 | 4 (31) |
| ORR (CR + CRi + PR) | 0 | 0 | 0 | 1 (50) | 4 (80) | 5 (71) | 5 (38) |
| **Higher-risk MDS** | ***N = 0*** | ***N = 2*** | ***N = 2*** | ***N = 1*** | ***N = 3*** | ***N = 4*** | ***N = 6*** |
| Best overall response, *n* (%) |  |  |  |  |  |  |  |
| CR | 0 | 0 | 0 | 0 | 0 | 0 | 0 |
| PR | 0 | 0 | 0 | 0 | 0 | 0 | 0 |
| HI | 0 | 0 | 0 | 0 | 0 | 0 | 0 |
| mCR | 0 | 0 | 0 | 1 (100) | 0 | 1 (25) | 1 (17) |
| SD | 0 | 2 (100) | 2 (100) | 0 | 2 (67) | 2 (50) | 4 (67) |
| Relapse after CR or PR | 0 | 0 | 0 | 0 | 0 | 0 | 0 |
| Cytogenic CR | 0 | 0 | 0 | 0 | 0 | 0 | 0 |
| PD^b^ | 0 | 0 | 0 | 0 | 1 (33) | 1 (25) | 1 (17) |
| ORR (CR + PR + HI) | 0 | 0 | 0 | 0 | 0 | 0 | 0 |

AML, acute myeloid leukemia; CR, complete remission; CRi, complete remission with incomplete blood count recovery; HI, hematologic improvement; mCR, marrow complete remission; MDS, myelodysplastic syndromes; ORR, objective response rate; PD, progressive disease; PR, partial remission; SD, stable disease

^a^All patients who received at least one dose of study drug, had a baseline disease assessment, and had at least one post-baseline disease assessment

^b^PD was assessed by investigator judgement

**Supplementary Fig. 1** Schematic of dosing cohorts in the study


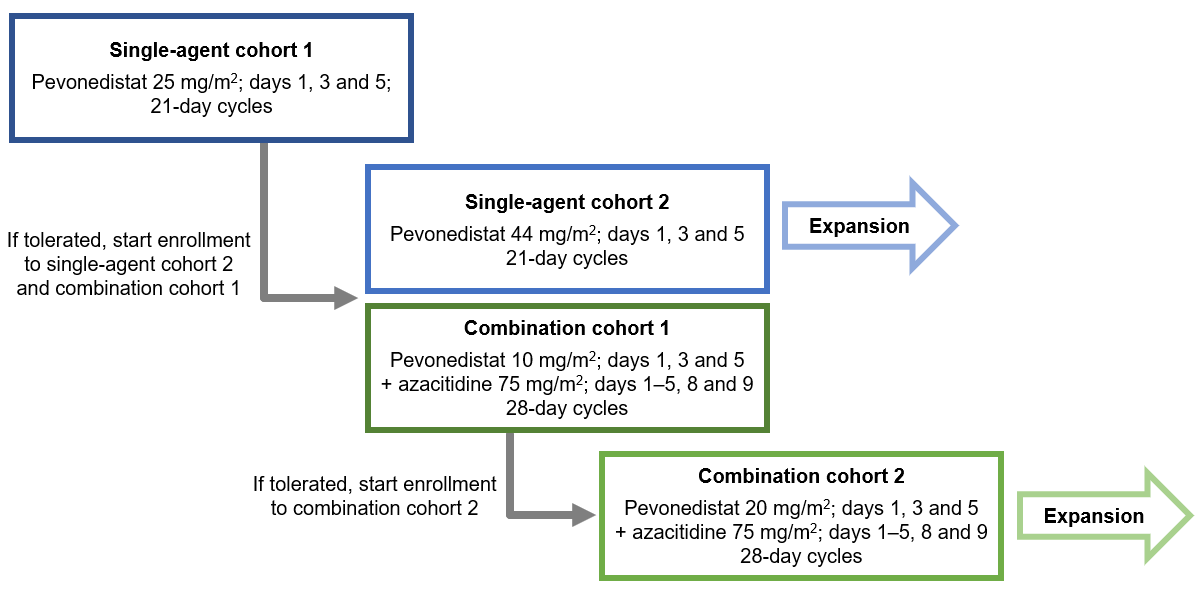

Supplement: Supplementary file 1 — Additional file 1. Supplementary methods, tables, and figure. [file 13045_2022_1264_MOESM1_ESM.docx]
